# The other side of recovery: validation of the Portuguese version of the subjective experiences of psychosis scale

**DOI:** 10.1186/s12888-015-0634-3

**Published:** 2015-10-14

**Authors:** Filipa Martins, Sandra C. Soares, Pedro Bem-Haja, Carolina Roque, Nuno Madeira

**Affiliations:** Department of Education, University of Aveiro, Aveiro, Portugal; Center for Health Technology and Services Research (CINTESIS-UA), Department of Education, University of Aveiro, Aveiro, Portugal; Karolinska Institute, Department of Clinical Neurosciences, Division of Psychology, Stockholm, Sweden; IBILI - Institute for Biomedical Imaging and Life Sciences, Faculty of Medicine, University of Coimbra, Coimbra, Portugal; Psychiatry Department, Coimbra Hospital and University Centre, Coimbra, Portugal; Department of Psychological Medicine, Faculty of Medicine, University of Coimbra, Coimbra, Portugal

**Keywords:** Psychosis, Schizophrenia, Bipolar disorder, Recovery, Self-report questionnaire

## Abstract

**Background:**

The aim of this study was to develop and validate a Portuguese version of The Subjective Experiences of Psychosis Scale (SEPS) for use in Portuguese-speaking populations in order to provide a self-report instrument to assess and monitor dimensions of psychotic experiences, translating patient’s perspective and experience in terms of recovery from psychosis.

**Methods:**

The sample consisted of 30 participants with psychotic disorders who had recently experienced delusions or hallucinations. The SEPS was completed along with other observer-based assessments and self-report questionnaires, such as the Brief Psychiatric Rating Scale, the Insight and Treatment Attitudes Questionnaire and the Function Assessment Short Test.

**Results:**

Two main factors representing the positive and negative components of each subscale were identified. We obtained good internal consistency and test-retest reliability for the positive and negative components of all subscales. The subscales of SEPS correlated with observer-based assessments and self-report questionnaires.

**Conclusions:**

The Portuguese version of the SEPS is a useful tool in the assessment and monitoring of psychotic symptoms.

## Background

Many instruments have been developed to assess psychotic symptoms and treatment effects. Such instruments have proven to be of interest in clinical practice and research because they allow for better symptom monitoring, treatment adaptation, and general improvement in the quality of mental health services. Most of these measures are observer assessments conducted by mental health professionals through interviewing and patient observation [1]. On the other hand, self-report questionnaires are based on patients’ perception of their mental health and treatment, without interference from mental health professionals and are, therefore, regarded as measures of subjective evaluation [2]. Self-report instruments - taking into account limitations to their use in psychotic patients such as lack of insight, cognitive deficits, and even metacognitive skills - have several advantages over observer assessments, namely their ease of use [3, 4]. They can also provide real-life information on perceived effects of treatment, which can only be described by the patient [2]. However, most of these instruments focus only on presence and severity of symptoms and do not take into account other dimensions that are relevant for patients, such as the positive and negative effects of the treatment [1]. Factors influencing subjective response to antipsychotic drugs include insight, previous experience of medication, health beliefs and the quality of the therapeutic relationship, and it is now widely accepted that patients’ subjective experience of medication should be given more consideration in treatment outcome measures [5].

The patient’s perspective is of particular importance in cases of severe mental illness where multiple areas may be affected. While some patients may value the reduction of symptoms or side effects, others may view social relations or global functioning as more important. Thus, two individuals with identical scores in a set of instruments focused only on psychopathological symptoms may have very different feelings towards their mental illness [6]. More importantly, a traditional perspective that psychotic disorders are associated with lack of insight – attributable to cognitive deficits or adaptation to disease, avoiding painful realities – should acknowledge that awareness of illness is intertwined with a person’s narrative understanding of his own life [7, 8].

The definition of recovery in psychosis has been addressed differently, depending on whether it is seen from a personal or clinical perspective. The clinical perspective is measured in terms of alleviation of symptoms associated with an improvement [9]. This view has been widely adopted [10] since it has been shown that psychotic symptoms contribute to a marked disruption in social and occupational functioning [11]. Consequently, a large number of instruments for measuring symptoms and functioning have been developed with high levels of reliability, reinforcing the idea of recovery as the reduction of psychiatric symptoms [12]. Such instruments have proven to be of interest in clinical practice and research because they allow a better symptom monitoring, treatment adaptation and general improvement in the quality of mental health services. However, most of these instruments focus on presence and severity of symptoms only, and do not take into account the multidimensional process of recovery as reported by patients [1, 13].

Although the classical hallmark of schizophrenia is the deteriorating course and its negative impact in patient’ lives, it is currently accepted that recovery is possible even for those individuals [14]. In fact, studies have shown diverse prognostic patterns in the first episode of psychotic patients [15]. The personal perspective of recovery occurs as an evolving and adjusting process through which the subject overcomes main challenges such as threats to its sense of identity, loss of previous ways of understanding the world, acknowledging losses in one’s life and accepting oneself as an ordinary yet proactive individual, achieving self-determination and a meaningful life [9, 16]. This does not necessarily mean a decline in psychiatric symptoms but rather the achievement of specific recovery goals defined by the patient [17]. In mental health policy this definition highlighted the importance of taking into account the patient’s subjective experiences, needs and preferences in evaluation and treatment [18]. However, its inherent subjectivity has made it more difficult to measure recovery adequately and to build these instruments [19].

Wood and cols [13] attempted to ascertain which factors elicited by patients contributed to recovery and how those related to a reduction in symptoms. Four main themes emerged: impact on mental health, individual change and adaptation, social redefinition, and development of coping strategies. Moreover, a similar and more recent study [20] identified further perspectives: collaborative support and understanding; emotional change through medical and social support; increased functionality and attainment of occupational goals; and recovery focused on individual factors. In both studies the importance of reduction of psychotic symptoms was taken into account for some patients whilst for others this concern was displaced for the absence of their negative impact, rather than their presence. Importantly, recovery seems to be interpreted as a multidimensional process and, therefore, the conceptualization of recovery must consider both symptoms and personal factors [10]. Studies have emphasized the importance of developing instruments that explore both clinical and personal aspects of recovery [12]. However, most of these studies have focused on each component individually, warranting the development of tools that measure recovery in a holistic fashion.

The Subjective Experiences of Psychosis Scale (SEPS) [1] has emerged from the need to provide a self-report scale of psychotic experiences, which would reflect the various dimensions of psychosis and allow the evaluation of treatment effects over time. In order to translate the experience and perspective of patients on recovery, the conception of the SEPS followed two distinct phases. In the first phase, items of the scale were generated by identifying in the literature relevant factors for the conceptualization of recovery from the patient’s perspective. In the second phase, patients with a history of psychosis were asked to put forward suggestions on the content and format of the scale. Forty-one items were derived and then organized into three categories: (1) impact of psychotic experiences in the mental health and well-being (29 items); (2) impact of support (treatment, social support, substance abuse, and religious beliefs) in psychotic experiences (6 items); and (3) severity of the different dimensions of psychotic experiences (6 items).

Participants are instructed to rate the impact of their experiences of psychosis during the previous week. Each item in the subscales 1 and 2 is listed twice for the positive and negative impact. For example, in the first item of the first subscale the following question is asked: “In the past week, how have your experiences affected your ability to socialize?”. Participants are instructed to rate both the positive and negative aspects of their ability to socialize, while using a five-point Likert scale, varying from (1) “Not at all” to (5) “Very Much”. For subscale 2, items can be quoted as “not applicable” if the aspect in question has not verified. The SEPS showed good internal consistency and test-retest reliability and demonstrated good validity against other measures of psychosis, appearing as a valid and reliable self-report scale to assess and monitor dimensions of psychotic experiences [1].

The aim of our study was to translate, adapt and validate a Portuguese version of SEPS [1] in order to provide a multidimensional scale to assess and monitor dimensions of psychotic symptoms. We intended to determine the internal consistency, reliability, validity and applicability of this instrument in the Portuguese population. The study hypotheses were that the scale presented good internal consistency (α of at least 0.6), reliability in test-retest, and good validity in relation to other measures of psychosis (criterion validity). More specifically, we expected that the negative components of the subscales of SEPS were positively correlated with measures of symptoms (general symptoms, functioning, depression, anxiety), and negatively correlated with recovery and that the positive components of SEPS were positively correlated with recovery and negatively correlated with measures of symptoms. Following previous studies of predictors of recovery using more traditional definitions, focused on symptom remission or non-recurrence, we also addressed relevant psychosocial and neuropsychiatric factors, such as insight and treatment adherence [21].

## Methods

### Participants

This project was approved by the Ethics Committee of the Faculty of Medicine, University of Coimbra (REF 12-CE-14). Patients were recruited from the outpatient clinic of the Psychiatry Department of the Coimbra Hospital and University Centre. After describing the aims and procedures of the study and ensuring confidentiality, participants were invited to voluntarily participate in the study. All individuals signed written informed consent before any procedure.

Patients were included in the study if they met the following inclusion criteria: (1) diagnosis of schizophrenia or other psychotic disorder (schizophreniform disorder, schizoaffective disorder, delusional disorder, brief psychotic disorder and substance-induced psychotic disorder), or bipolar disorder type I (with at least one episode with psychotic features) based on the DSM-IV-TR criteria [11]; (2) recent experience (previous month) of delusions or hallucinations verified by the Brief Psychiatric Rating Scale (BPRS; [22]); (3) clinical capability to conscientiously understand the purpose and procedures of this research project and directly written informed consent; (4) aged between 18 and 65.

Exclusion criteria included: (1) inability to complete assessment questionnaires; (2) poor command of the Portuguese language; (3) severe cognitive deterioration; (4) neurological disorders; (5) comorbid drug or alcohol abuse or dependence. The study was approved by the local ethics committee and informed consent was obtained from all participants.

The sample consisted of 30 participants (10 females and 20 males) aged between 18 and 63 years (M = 38.27, SD = 11.87). Demographic information can be found in Table 1. All patients were medicated with psychotropic drugs: 12 with atypical and 7 with typical antipsychotics, 3 with both typical and atypical antipsychotics, and 8 with mood stabilizers and antipsychotics.

**Table 1:** Demographic information of the sample

Variable	Number (%)
Gender	
Male	20 (66,7)
Female	10 (33,3)
Age, Mean (SD)	38,3 (11,9)
Marital status	
Single	18 (60,0)
Married	8 (26,7)
Divorced	4 (13,3)
Household	
Alone	2 (6,7)
Parents	16 (53,3)
Partner	8 (26,7)
Friends	3 (10,0)
Institution	1 (3,3)
Education level	
Basic	7 (23,3)
Secondary	14 (46,7)
Higher	9 (30,0)
Employment status	
Employed	13 (43,3)
Unemployed	9 (30,0)
Student	2 (6,7)
Home worker	1 (3,3)
Retired	5 (16,7)
Diagnosis	
Schizophrenia	15 (50)
Schizoaffective disorder	2 (6,7)
Brief psychotic disorder	2 (6,7)
Bipolar disorder type I	11 (36,7)
Duration of disorder, mean years (SD)	
	7,70 (7,72)

### Instruments

A structured questionnaire was used to collect demographic information. Three observer assessments were used: the Brief Psychiatric Rating Scale to rate overall psychiatric symptoms (BPRS; [23]), the Insight and Treatment Attitudes Questionnaire to assess insight and attitudes towards treatment (ITAQ; [24]) and the Portuguese version of the Function Assessment Short Test to measure general functioning [25] (FAST; [26]).

The SEPS and four other self-report questionnaires were used: the Recovery Assessment Scale (RAS; [27]) was used to ascertain how the SEPS related to reported levels of recovery; the Portuguese version of the Brief Symptom Inventory [28] (BSI, [29]) was used to assess overall psychiatric symptoms; the Portuguese version of the Beck Depression Inventory (BDI-II; [30]) [31], was used to assess depressive symptoms. Medication adherence was verified by the Portuguese version of the Medication Adherence Rating Scale [32] (MARS; [33]). All scales have shown to have good reliability and validity in samples of patients with psychosis.

### Procedure

To ensure content validity the original version was translated and adapted into Portuguese language by three Portuguese mental health professionals with proficiency in English. All versions were compared and modified until a consensus version was agreed upon. Back-translation to English was then performed by a bilingual mental health professional with no prior knowledge of the scale. The back-translation was sent to the authors of the original scale for revision and official approval of the Portuguese version.

Participants were recruited from the outpatient clinic of the Psychiatry Department at the Coimbra Hospital and University Centre, a tertiary care centre. The study was conducted over two separate phases. In phase one, the demographic questionnaire and all scales were completed. The ITAQ and the FAST interviews were carried out by the first author and the BPRS was conducted by the referring psychiatrist and then reviewed by one of the authors. All remaining scales were completed independently by participants, with no interference from mental health professionals.

In order to investigate test-retest reliability of the Portuguese version of the SEPS, a second phase was conducted 3–4 weeks later, in which participants were asked to complete the SEPS again. Eighteen participants completed this phase, given that 12 patients were unavailable to return for re-evaluation.

### Statistical analysis

Statistical analysis was performed using the software SPSS Statistics 21 (IBM Corporation, Armonk, NY, USA). Faced with the normal distribution of the sample data, one-way ANOVAS, t-tests and Pearson’s r were performed to explore some differences and relationships between the variables in use. This study aims to validate a scale and, therefore, an exploratory factor analysis (PCA method) was performed to assess the construct validity. Pearson’s correlations between the SEPS and other competing/comparison measures were accomplished to assess criterion validity the Cronbach’s alpha was used to calculate internal consistency. Pearson correlation and Intraclass Correlation Coefficient (ICC) were used to assess reliability test-retest.

## Results

Thirty participants (10 females and 20 males) were enrolled in the study and their demographic characteristics are summarized in Table 1. All 30 participants completed phase one of the study but only 18 completed the second phase.

Due to the limited number of participants in the sample, the “not applicable” option in subscale 2 (“Impact of Support in Experiences”) had a negative impact on the results, since these data were not taken into account in the statistical analyses. The items listed comprised: “Support from other service users”, “Alcohol and/or drug use” and “Spirituality/Religious Beliefs”. To address this lack of data, the tendency to response to items model could be taking into account. However, due to the specificity of each item, the cold deck imputation method was preferred, and the missing values were replaced by the mean of the other participant’s responses in the three questions [34].

### Descriptive analysis

Descriptive analyses were conducted to explore the socio-demographic and clinical characteristics of the sample. Table 2 presents descriptive statistics for the measures of symptomatology, insight and treatment adherence for each of the diagnoses in the sample. In general, patients with a diagnosis of schizophrenia presented higher scores in general symptoms (BPRS), and lower levels of insight (ITAQ) and recovery (RAS). Patients with bipolar disorder showed higher levels of insight and treatment adherence (MARS). Functionality levels (FAST) were lower for schizoaffective and bipolar patients. A one-way ANOVA, performed to compare the means between the different diagnoses, showed that none of the differences were significant.

**Table 2:** Descriptive statistics of instruments for each diagnosis

Diagnosis	BPRS mean (SD)	ITAQ mean (SD)	FAST mean (SD)	RAS mean (SD)	MARS mean (SD)
Schizophrenia	39.20 (8.39)	14.92 (6.90)	15.38 (10.71)	152.00 (22.63)	6.85 (2.27)
Schizoaffective	35.50 (4.95)	19.50 (3.54)	19.50 (6.36)	152.93 (18.68)	6.50 (0.71)
Brief Psychotic	32.50 (2.12)	18.00 (1.41)	13.00 (9.90)	166.00 (8.49)	6.50 (0.71)
Bipolar I	37.36 (7.97)	19.60 (4.06)	19.20 (17.93)	157.18 (11.33)	7.10 (2.03)

*SD* standard deviation

A Student’s *t*-test for independent samples was used to determine whether there were significant differences between gender and the scores obtained in each subscale of the SEPS. Gender differences in the various subscales were small, and men ranked higher in SEPS’s negative aspects. The only significant difference found was for the subscale “Positive Impact of Support in Psychotic Experiences”, *t* (28) = 3.45, *p* = 0.002, showing that women identified a greater positive impact of support on their experiences (M = 21.40, SD = 2.17) than men (M = 10.17, SD = 3.61).

A one-way ANOVA was performed to check whether there were significant differences among the diagnoses and the scores on each of the subscales of SEPS (Table 3). No statistically significant differences were observed. The descriptive statistics for each of the subscales of SEPS in both assessment stages, as well as for gender and diagnoses separately, are presented in Table 3. Only the responses provided by the participants in the first phase of the assessment were used for the analysis of gender and diagnosis.

**Table 3:** Descriptive statistics for the subscales of SEPS and for gender and diagnosis

	Phase 1			Phase 2
Subscale	Total (*n* = 30)	Male (*n* = 20)	Female (*n* = 10)	Total (*n* = 18)
	Mean (SD)	Mean (SD)	Mean (SD)	Mean (SD)
Positive impact	71.47 (24.32)	73.85 (26.15)	66.70 (20.58)	66.56 (21.62)
Negative impact	62.97 (21.26)	64.00 (21.03)	62.45 (21.91)	60.17 (20.43)
Positive support	18.53 (3.78)	17.10 (3.61)**	21.40 (2.17)**	16.28 (3.88)
Negative support	9.37 (3.07)	9.80 (3.38)	8.50 (2.22)	7.67 (2.97)
Positive dimensions	4.73 (2.32)	4.95 (2.37)	4.30 (2.26)	4.33 (1.64)
Negative dimensions	9.40 (3.59)	9.75 (3.71)	8.70 (3.40)	9.78 (3.25)
Subscale	Schizophrenia	Brief Psychotic	Bipolar I	Schizoaffective
	Mean (SD)	Mean (SD)	Mean (SD)	Mean (SD)
Positive impact	73.07 (24.90)	67.00 (15.56)	71.45 (24.32)	64.00 (14.14)
Negative impact	60.20 (22.27)	73.50 (10.61)	65.18 (23.37)	61.00 (14.14)
Positive support	16.93 (4.16)	23.00 (1.41)	19.27 (2.28)	22.00 (2.83)
Negative support	9.40 (3.20)	10.50 (2.12)	8.91 (2.77)	10.50 (6.36)
Positive dimensions	4.93 (2.37)	4.00 (2.83)	4.82 (2.44)	3.50 (2.12)
Negative dimensions	8.67 (3.39)	8.00 (4.24)	11.00 (3.66)	7.50 (3.54)

*SD* standard deviation

**p <0.01

In the analysis of correlations between the positive and negative components of each subscale, there was a significant positive correlation between the positive component of subscale “Impact of Experiences” and the positive component of the subscale “Dimensions of Experiences”. The effect size of this correlation was large (r = 0.72, *p* = 0.000). The negative component of the subscale “Impact of Experiences” showed a significant positive correlation with the negative component of the subscale “Impact of Support in Experiences”, and with the negative component of subscale “Dimensions of Experiences”, also both with a large effect size (r = 0.57, *p* =0.001 and r = 0.50, *p* =0.005 respectively) (see Table 4).

**Table 4:** Correlations between subscales of SEPS

Subscale	Positive impact	Negative impact	Positive support	Negative support	Positive dimensions	Negative dimensions
Positive impact	–	–	–	–	–	–
Negative impact	0.13	–	–	–	–	–
Positive support	0.25	0.30	–	–	–	–
Negative support	0.34	0.57**	0.10	–	–	–
Positive dimensions	0.72**	−0.12	0.21	0.09	–	–
Negative dimensions	0.19	0.50**	0.05	0.31	0.21	–

**p <0.01

### Factor analysis

For the purpose of determining whether the Portuguese version of SEPS shows the same factor solution (two factors for each subscale - positive and negative) than that of the original validation study (1), we decided to run an exploratory factor analysis with the PCA method. In order to verify this separation of factors, a principal component analysis was applied to each subscale of the SEPS, using a varimax rotation. Due to the clinical nature of the sample, the number of participants was limited, thus involving constraints in the factor analysis. However, in order to verify the suitability of the data for conducting a factor analysis, the correlation matrix was examined to ensure that there was no multicollinearity or singularity. The Kaiser-Meyer-Olkin (KMO) coefficients were also explored to verify the adequacy of the sample [35]. The coefficient for the first subscale (“Impact of Experiences”) was weak, yet acceptable (KMO = 0.50). Both the second (“Impact of Support in Experiences”) and third subscales (“Dimensions of Experiences”) yielded moderate coefficients (KMO = 0.60 and KMO = 0.63 respectively). The values for the Bartlett test (29) were significant for the three subscales (*p* <0.05). As it happened in the original study, for each of the three analyses two main factors emerged (for eigenvalues higher than 1 and loadings above 0.4 taken as significant). For the first subscale, two factors contributed to 36.50 % of explained variance (40.13 %, in the original study), with factor 1 (Positive Impact of Experiences) contributing to 18.34 % of the variance (21.54 %, in the original study) and factor 2 (Negative Impact of Experiences) contributing to 18.16 % of the variance (18.59 %, in the original study). For the second subscale two factors that explain 40.69 % of the variance (54.29 %, in the original study) were identified. Factor 1 (Positive Impact of Support in Experiences) contributed to 21.59 % of the variance (29.49 %, in the original study) and factor 2 (Negative Impact of Support in Experiences) to 19.11 % of the variance (24.80 %, in the original study). For the third subscale, two factors contributed to a total explained variance of 75.40 % (60.12 %, in the original study). Factor 1 (Positive Dimensions of Experiences) contributed to 45.67 % of the variance (33.47 %, in the original study) while the second factor (Negative Dimensions of Experiences) contributed to 29.73 % of the variance (26.65 %, in the original study).

### Reliability and internal consistency

To explore the reliability of SEPS, the Cronbach’s alpha was used for the calculation of internal consistency of each subscale and the Pearson correlation coefficient (r) and ICC to ascertain the test-retest reliability between the two administrations.

Regarding internal consistency, excellent values were obtained for the positive and negative components of the subscale “Impact of Experiences” (both with α = 0.96). The values for the positive and negative components of the subscale “Impact of Support in Experiences” were acceptable (α = 0.61 and α = 0.67 respectively), and for positive and negative components of the subscale “Dimensions of Experiences” the values ranged from excellent to good (α = 0.92 and α = 0.84 respectively). After the analysis of the correlation matrix for each subscale, which relates each item to the total correlation of the test, it was found that there was no item that if eliminated would significantly increase the internal consistency of a subscale (see Table 5).

**Table 5:** Measures of reliability: intern consistency and test-retest reliability

Subscale	Internal consistency Cronbach α	Test-retest reliability Pearson / ICC
Positive impact	0.96	0.93**/ 0.962
Negative impact	0.96	0.84**/ 0.903
Positive support	0.61	0.52*/ 0,557
Negative support	0.67	0.72**/ 0,783
Positive dimensions	0.92	0.66**/ 0.787
Negative dimensions	0.84	0.77**/ 0,857

**p <0.01; *p <0.05

For the test-retest reliability assessment, the values of Pearson coefficients were statistically significant for the positive and negative components of each subscale and the size effect was large for all of them (*r* = 0.93, *p* <0.01 and *r* = 0.84, *p* <0.01 for the components of the subscale “Impact of Experiences”, *r* = 0.52, *p* <0.05 and *r* = 0.72, *p* <0.01 for the components of the subscale “Impact of Support in Experiences”, *r* = 0.66, *p* <0.01 and *r* = 0.77, *p* <0.01 for the components of the subscale “Dimensions of Experiences”) (see Table 5). In addition, and in order to deepen the analysis of test-retest reliability, we decided to calculate the Intraclass Correlation Coefficient (ICC) using the absolute agreement method (see Table 5).

### Correlation analyses

The criterion validity of SEPS was verified using bivariate correlation analysis and through the Pearson correlation coefficient (r). We obtained internal consistency values considered appropriate for BPRS, FAST, BSI, RAS and BDI-II (α = 0.78, α = 0.94, α = 0.98, α = 0.85 and α = 0.86 respectively).

The Global Severity Index (GSI) of BSI showed significant positive correlations of large effect size with the negative components of the subscales of “Impact of Experiences” and “Impact of Support in Experiences”, and of average effect size with the negative component of the subscale “Dimensions of Experiences” (*r* = 0.74, *p* <0.01, *r* = 0.52, *p* <0.01 and *r* = 0.41, *p* <0.05 respectively). The BDI-II correlated negatively with the positive components of the subscales and positively with the negative components of the subscales. A significant positive correlation of large effect size was founded between the BDI-II and the negative component of the subscale “Impact of Experiences” (*r* = 0.53, *p* <0.01).

The RAS correlated positively with the positive components of the subscales and negatively with the negative components of the subscales. These correlations were significant and with an average effect size for the positive components of subscales “Impact of Experiences” (*r* = 0.40, *p* <.05), “Impact of Support in Experiences” (*r* = 0.39, *p* <0.05) and “Dimensions of Experiences” (*r* = 0.40, *p* <0.05), and for the negative component of subscale “Impact of Experiences” (*r* = -0.43, *p* <0.05).

The dimensions Anxiety and Psychoticism of the BSI were significantly correlated with all the negative components of the subscales of SEPS and the effect size ranged from large to average (*r* = 0.75, *p* <0.01 and *r* = 0.55, *p* <0.01 respectively, for the subscale “Impact of Experiences”, *r* = 0.45, *p* <0.05 and 0.55, *p* <0.01 respectively, for subscale “Impact of Support in Experiences”, *r* = 0.41, *p* <0.05 and *r* = 0.39, *p* <0.05 respectively, for the subscale “Dimensions of Experiences”) (see Tables 6 and 7).

**Table 6:** Correlations between the subscales of the SEPS and other measures of symptoms and recovery

Subscale	GSI (BSI)	RAS	BDI-II	Anxiety (BSI)	Psychoticism (BSI)
Positive impact	0.17	0.40*	−0.10	0.13	0.37
Negative impact	0.74**	−0.43*	0.53**	0.75**	0.55**
Positive support	0.04	0.39*	−0.01	0.14	0.06
Negative support	0.52**	−0.12	0.13	0.45*	0.55**
Positive dimensions	−0.20	0.40*	−0.12	−0.21	0.07
Negative dimensions	0.41*	−0.20	0.35	0.41*	0.39*

*p <.05; **p <.01

**Table 7:** Correlations between the subscales of the SEPS and other measures of symptoms and recovery

Subscale	BPRS total	FAST	Depression (BPRS)	Anxiety (BPRS)
Positive impact	0.21	−0.29	−0.20	0.18
Negative impact	0.24	0.44*	0.47**	0.41*
Positive support	0.13	−0.13	0.20	0.28
Negative support	0.16	0.22	0.18	0.10
Positive dimensions	0.20	−0.23	−0.16	0.17
Negative dimensions	0.51**	0.14	0.45*	0.50**

*p <.05; **p <.01

The BPRS showed a significant positive correlation of large effect size with the negative component of the subscale “Dimensions of Experiences” (*r* = 0.51, *p* <.01). The FAST correlated negatively with the positive components of the subscales and positively with the negative components subscales. There was a significant correlation, of average effect size, between FAST and the negative component of the subscale “Impact of Experiences” (*r* = 0.44, *p* <0.05). The Depression item of the BPRS was significant correlated with the negative components of the subscales “Impact of Experiences” (*r* = 0.47, *p* <0.01) and “Dimensions of Experiences” (*r* = 0.45, *p* <0.05), and these correlations showed an average effect size. The Anxiety item of the BPRS showed significant positive correlations, with an effect size ranged from average to large, with the negative components of the subscales “Impact of Experiences” (*r* = 0.41, *p* <0.05) and “Dimensions of Experiments” (*r* = 0.50, *p* <0.01) (see Tables 6 and 7).

### Applicability

The applicability of the scale was determined by calculating the proportion of participants who did not provide answers to all questions of the scale. As this did not happen, applicability of the SEPS was 100 %.

The participants took an average of 30–45 min to complete the scale. While using a five-point Likert Scale, 22 participants (73.3 %) reported that they experienced no distress when completing the scale, 5 (16.7 %) reported feeling “a little” distress and only 3 (10 %) reported experiencing “moderate” distress.

## Discussion

The aim of this work was to validate the SEPS for the Portuguese population and provide to Portuguese mental health professionals an instrument to assess and monitor the dimensions of psychotic symptoms and actual effects of treatment in individuals. Like the original questionnaire, the validation of the European Portuguese version of SEPS started with the formulation of detailed definitions of the construct, derived from medical and psychological heuristics and supported by previous research regarding systematic observation and analysis of the relevant domains of behavior. Subsequently the Portuguese version of SEPS underwent a psychometric analysis to determine its validity, reliability and applicability.

### Validity assessment

To examine validity, we used the Trinitarian model adopted by the American Psychological Association, which divides the validity of a psychometric instrument into content validity, criterion validity and construct validity.

### Content validity

Regarding content validity, procedures such as spoken reflection and independent back-translation required in psychometric practice were followed. These processes ensured that the test items do not undergo semantic and conceptual deviations from the original scale and are prepared to fit to the definitions of the construct. The Portuguese version of SEPS also provides a representative sample of a finite universe of behaviors (Subjective Experiences of Psychosis).

### Criterion validity

After ensuring the content validity of the Portuguese version of SEPS, we looked at whether the assessed construct is subject to appropriate behavioral representation, checking if the two factors that are defined theoretically can be replicated with the empirical data from our sample. For this we carried out a factor analysis using the principal components method, and adopting the eigenvalues and minimum correlation coefficients used by the authors in the original scale [1]. As expected, there was a clear division between positive and negative dimensions (two factors). This also happened in the validation study of the original version of SEPS [1]. This result ensured that the Portuguese version of SEPS is a legitimate and appropriate representation of the measured theoretical construct, i.e., it has construct validity.

### Concurrent validity

We also intended to determine whether the Portuguese version of SEPS is effective to predict the specific performance of a subject. The subject’s performance was assessed concurrently (concurrent validity) through independent scales already validated for the Portuguese population that assess constructs or concepts present in SEPS. In general, the negative dimensions of SEPS were positively correlated with general measures of symptoms (BPRS, BSI), functionality (FAST), depression (BPRS, BDI-II), anxiety (BPRS, BSI) and psychoticism (BSI), and negatively correlated with the measure of recovery (RAS).

The positive dimensions of SEPS, despite positive correlations with the measure of recovery, were not always negatively correlated with measures of symptoms. That is in line with previous findings describing no direct correlation between observer ratings of symptoms and subjective self-report of being in recovery [17]. Such occurrence is also not surprising when considering that some positive symptoms are hypothesised to shield people from low self-esteem, in line with the so called ‘protective’ function of a lack of insight in psychosis [8, 36]. In fact, it has been identified that, in schizophrenia patients, increase in self-awareness and insight is a risk factor for depression and suicide [16, 37]. Additionally, the awareness of symptoms has been found to be influenced by an individual’s coping style, with patients unaware of their symptoms having a greater preference for positive reappraisal [38].

All instruments used in the criterion validity of SEPS had, in our sample, internal consistency values considered appropriate. Thus, we can say that the Portuguese version of SEPS shows criterion-related validity.

### Reliability

To assess the reliability of the Portuguese version of SEPS we verified its temporal constancy measured by test-retest and internal consistency using Chronbach’s alpha. Using DeVellis parameters [39] internal consistency values considered appropriate for the positive and negative components of subscales of “Impact of Experiences” and “Dimensions of Experiences” (α >0.80), and acceptable values (α >0.6) for the positive and negative components of the subscale “Impact of Support in Experiences” were obtained. The lowest values of Cronbach’s alpha obtained for the subscale “Impact of Support in Experiences” can be explained by the different dimensions assessed by this subscale. In the remaining subscales, negative parameters were usually associated with the presence of symptoms and positive at higher levels of recovery, thereby obtaining good correlations between the positive and the negative items from these subscales. This lends support to the good levels of internal consistency found. However, in the Subscale “Impact of Support in Experiences” the positive and negative items are not necessarily interrelated because they refer to aspects (medication, social support, use of alcohol or drugs and religious beliefs) that are not directly associated with the symptoms. This may explain the opposite responses from a participant. The test-retest reliability proved to be very good for all components of the subscales of SEPS. Thus, with regard to reliability, the Portuguese version of SEPS has good internal consistency and very good temporal constancy.

### Applicability

All participants responded to all the questions of scale, i.e., the Portuguese version of SEPS has 100 % of applicability.

### Limitations and strengths

The major limitations of this study are the small sample size given the scarcity of subjects that accepted inclusion in the study, the deteriorated clinical condition of some patients compromising the collection of data and the drop-outs.

The inclusion of patients presenting different diagnosis along the psychotic spectrum (schizophrenia, schizoaffective, brief psychotic disorder and bipolar disorder with psychotic features), represent a major strength of the Portuguese version of SEPS, expanding the applicability of its original version.

## Conclusions

From the analyses described above, despite a small sample size, we can assume that the European Portuguese version of SEPS has good psychometric properties and can be a useful scale for assessing and monitoring dimensions of psychotic symptoms. The Portuguese version of the SEPS may be useful to measure the recovery status of individuals during treatment, allowing the assessment of the negative impact of psychotic experiences, according to the patient’s own perspective. It also enables to tailor treatment to each particular individual (treatment, family, friends, *etc.*), allowing long-term and sustained improvement in the prognosis of patients with psychotic symptoms.
